# Damping and Mechanical Properties of Epoxy/316L Metallic Lattice Composites

**DOI:** 10.3390/ma16010130

**Published:** 2022-12-23

**Authors:** Yanpeng Wei, Huaiqian Li, Hao Yang, Yingchun Ma, Jingchang Cheng, Peng Gao, Jian Shi, Bo Yu, Feng Lin

**Affiliations:** 1 State Key Laboratory of Light Alloy Casting Technology for High-end Equipment, Shenyang Research Institute of Foundry, Shenyang 110022, China; 2 Department of Mechanical Engineering, Tsinghua University, Beijing 100084, China; 3 Key Laboratory of Space Physics, Beijing 100076, China

**Keywords:** epoxy/316L metallic lattice composites, high damping, mechanical properties, energy absorption

## Abstract

The lattice structure was prepared by selective laser melting of 316L metal powder, and the epoxy was naturally infiltrated into the pores of the 316L metallic lattice structure. The epoxy/316L metallic lattice composites with integrated structure and function were prepared. Scanning electron microscopy was used to observe the microstructure of the epoxy/316L metallic lattice composites. The damping performance of the epoxy/316L metallic lattice composites were studied by modal measurement method. At the same time, the engineering stress–strain curve was obtained by a quasi-static compression experiment on a universal testing machine. The results show that the interface of epoxy and 316L metallic lattice is well bonded, and there are a few bubbles in the epoxy. The epoxy/316L metallic lattice composites have high damping characteristics with damping ratio over 10%. The energy absorption of epoxy/316L metallic lattice composites is as high as 68.32 MJ/m^3^, showing high energy absorption characteristics.

## 1. Introduction

Vibration and noise reduction in aerospace, marine and other high-end equipment is a technical problem to be solved. The explosive growth of technology brings more possibilities to industry. Industrial equipment is becoming more and more high-speed and automatic, which also brings various levels of noise and vibration, which will reduce the accuracy of the equipment, and at the same time, staff may experience irritability and other related emotions, which will seriously affect their physical and mental health. Therefore, damping materials with the function of vibration and noise reduction have widely concerned researchers [1,2,3,4]. Damping materials in aerospace technology, ships and other high-end equipment often need to meet versatility requirements, not only need to have the function of noise reduction, but also the mechanical properties of structure. For example, the connection between the equipment and the hull is usually an elastic connection and a rigid connection. When the connection is elastic, the noise reduction effect is obvious, but the rigidity is too low, and the vibration intensity of the equipment installed on it is too high, which leads to a great potential safety hazard [4]. Elastic connections using mostly viscoelastic materials, such as silicone rubber, styrene butadiene rubber, etc. are prone to aging problems and need to be replaced frequently, increasing the cost of operation and maintenance [2]. When rigid connections are used, the materials used are mostly metals, such as Fe–Ga, NiTi and other alloys [5,6,7,8,9]. However, their damping ratio is basically 10^−3^ orders of magnitude, through the structural design can reach 10^−2^ orders of magnitude, but the distance is far less than the viscoelastic material at 10^−1^ orders of magnitude: safety is greatly improved but with almost no ability to reduce noise.

The damping performance of porous materials can improve the overall damping performance, as well as structural bearing capacity. The essence of porous materials allows it to obtain high damping characteristics through structural design and the material itself [10,11,12]. Researchers have developed a series of disordered porous materials, such as foamed aluminum, TiNiCu, etc. [13,14,15,16,17]. However, its damping ratio is usually not more than 3% with a low damping performance. There is still a big gap from the use, which cannot meet the requirements. Composites prepared by filling viscoelastic materials into porous materials have high damping characteristics, and their damping ratio is more than 10%, which indicates good damping characteristics. The filled viscoelastic materials include epoxy, rubber, polyurethane, etc. [18,19,20,21,22,23,24,25,26]. Although disordered porous materials have outstanding performance, it is difficult to achieve precise control and design due to process limitations, which brings great difficulties to practical applications. Ordered lattice metal is a better choice for structural bearings.

The lattice metal prepared by additive manufacturing technology is a lightweight material with high specific strength, stiffness and energy absorption capacity [27,28,29,30,31,32]. Through the design of 3D modeling software, it is possible to accurately create an orderly opening structure. 316L has good mechanical properties and is a cost-effective stainless steel. Therefore, the 316L metallic lattice structure is prepared by selective laser melting technology, and the natural seepage of epoxy is filled into the 316L metallic lattice structure to enhance the damping performance and mechanical properties of the material. The preparation of epoxy/316L metallic lattice composites is a solution with better sound reduction and structural bearing capacity. The interfacial bonding, damping properties, mechanical properties and energy absorption characteristics of epoxy/316L metallic lattice composites were studied.

## 2. Materials and Methods

### 2.1. Materials

**Figure 1:**
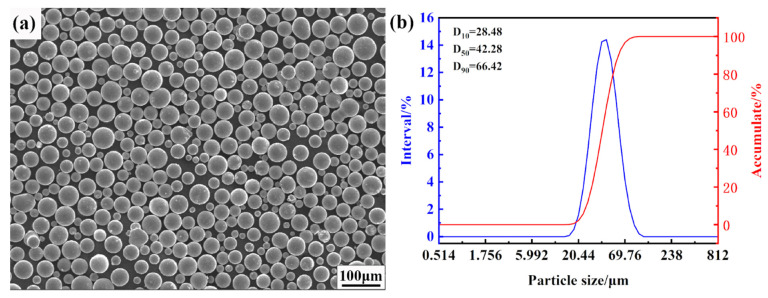
(**a**) Secondary electron micromorphology of 316L metal powder (SEM) and (**b**) particle size distribution of 316L metal powder.

316L metal powder (Shenzhen Micro-nano Additive Technology Co., Ltd., Shenzhen, China) was used as raw material in the present study. E51 epoxy and curing agent (Guangzhou Suixin Chemical Co., Ltd., Guangzhou, Chian) were mixed. Acetone (T, JKEMAO CHEMICAL REAGENTS Co., Ltd., Tianjing, China) was used to dilute to reduce viscosity; dibutyl phthalate (Shenyang New City Chemical Plant, Shenyang, China) was used to improve the curing toughness. Figure 1a shows the surface scanning electron microscopy image of 316L metal powder prepared by atomization technology. The powder is spherically and evenly distributed. The D50 of the powder, measured by laser particle size analyzer (BT-9300ST, Dandong better Co., Ltd., Dandong, China), is 42.28 μm in Figure 1b, which is normally distributed and suitable for additive manufacturing.

### 2.2. Experimental

The 316L metallic lattice structure was fabricated on a selective laser melting forming machine (BLT-310, Bright Laser Technologies Co., Ltd., Xi’an, China). Firstly, the forming chamber was vacuumed, and then high purity argon was injected to ensure that the oxygen content in the chamber was less than 100 ppm during the laser selective melting process. Laser scanning power was 220 W, scanning speed was 1000 mm/s, laser scanning spacing was 0.1 mm, powder thickness was 0.03 mm and scanning angle was 45°. The energy density E is calculated by Equation (1).

(1) $$E=\frac{P}{h\times d\times v}$$

whereby *P* is laser scanning power, *h* is laser scanning spacing, *d* is powder layer thickness and *v* is laser scanning speed. The calculated energy density is 73.3 J/mm^3^. The prepared samples were subjected to solid solution treatment at 1040 °C for 30 min and water cooling conditions to improve the properties. After heat treatment, the structure was placed in an ultrasonic cleaning device containing absolute alcohol (T, JKEMAO CHEMICAL REAGENTS Co., Ltd., Tianjing, China) for ultrasonic cleaning for 0.5 h to remove surface contaminants.

The E51 epoxy was weighed according to the mass, 15% DBP, 25%~30% curing agent, and stirred evenly by a magnetic stirrer. The utensils were placed in a digital display constant temperature water bath with a temperature of 50 °C and heated in the water bath; after 10 min, 5% diluent acetone was added, and it was subjected to stirred heating to achieve the best flow performance. The configured epoxy was poured into the 316L metallic lattice structure. The epoxy cured at room temperature for 24 h.

Scanning electron microscopy (Sigma 300, Zeiss, Germany) was used to observe the morphology and interface of the material, and a general universal testing machine (WDW-100) was used to test the quasi-static compression properties. The loading speed was 1 mm/min to study the mechanical behavior of the material. At the same time, the energy absorption characteristics of the material were characterized by energy absorption per unit volume and energy absorption efficiency. The stress–strain curve of the ideal energy-absorbing material has a constant stress, while the stress–strain curve of the actual energy-absorbing material has an elastic stage and a densification stage. Therefore, the higher the energy absorption efficiency, the closer the material is to the ideal energy absorption material. Equation (2) is the calculation formula of energy absorption C and per unit volume in the interval $\left[\varepsilon_{1},\varepsilon_{2}\right]$, and the energy absorption efficiency E(ε) of Equation (3) is used to evaluate the energy absorption characteristics of the structure.

(2) $$C=\int_{\varepsilon_{1}}^{\varepsilon_{2}}\sigma_{m}d\varepsilon$$

(3) $$E\left(\varepsilon \right)=\frac{\int_{\varepsilon_{1}}^{\varepsilon_{2}}\sigma_{m}d\varepsilon }{\sigma_{max}}$$

whereby $\sigma_{m}$ is stress, $\varepsilon_{m}$ is strain and $\sigma_{max}$ is the maximum stress in the interval $\left[\varepsilon_{1},\varepsilon_{2}\right]$.

**Figure 2:**
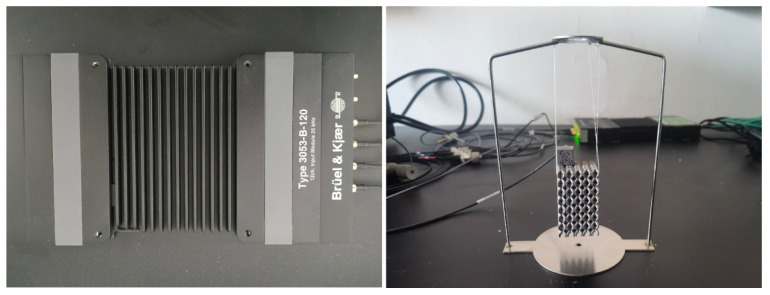
Model test equipment and specimen test.

Damping is one of the basic dynamic characteristics of the structure, and it is an important parameter to describe and reflect the energy dissipation characteristics in the process of structural vibration. The damping of the system was measured by the modal method, and the modal test of the system was analyzed by vibration and noise test (3053-B-120 Brüel&Kjær Co., Ltd., Lyngby-Taarbæk, Denmark). Figure 2 shows the experimental diagram of the damping ratio obtained by the modal test. The modal parameters were obtained by excitation and response, and the damping parameters of the material were calculated on the BK CONNECT operation software.

## 3. Results and Discussion

### 3.1. Damping Properties

**Figure 3:**
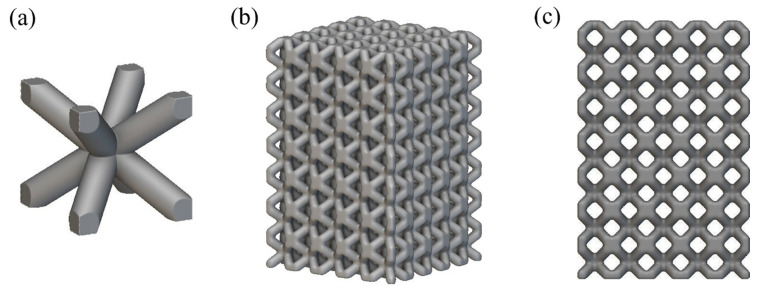
Lattice structure and main view of the lattice structure of the 3D-printed metallic lattice. (**a**) unit cell. (**b**) lattice structure. (**c**) main view of the lattice structure.

Figure 3 shows unit cell of the lattice structure and main view of the lattice structure of the 3D-printed metallic lattice. The unit cell of the lattice is a BCC structure with a side length of 6 mm. The diameter of the rod is 2 mm, and the center connection is smoothed. The size of the whole lattice structure is 30 mm × 30 mm × 45 mm. Figure 3c is the main view of the structure: the holes in the lattice can be clearly observed, and through actual measurement we can determine that the porosity is 56.59%, effectively reducing the weight.

**Figure 4:**
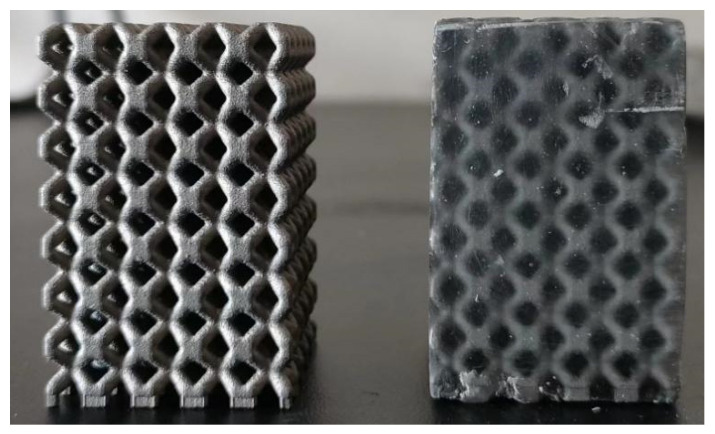
Fabrication of 316L metallic lattice structure and epoxy/316L metallic lattice structure.

In the actual printing, in order to eliminate the influence of additive manufacturing substrate, a 1 mm thick base should be added. Figure 4 is the fabrication of the 316L metallic lattice structure and epoxy/316L metallic lattice structure. Epoxy is fully filled into the lattice metal.

**Figure 5:**
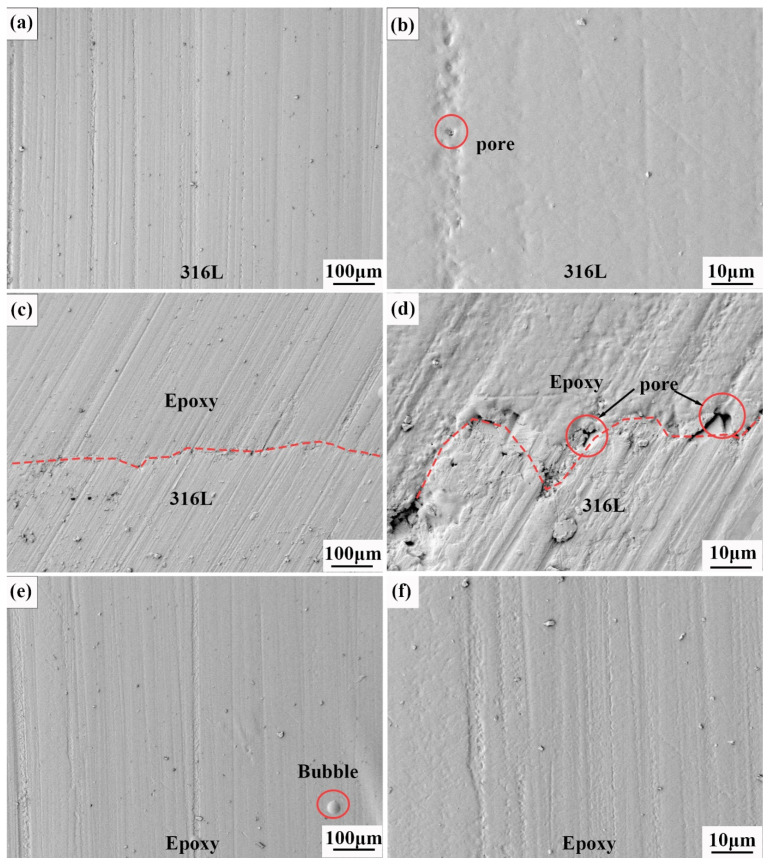
Secondary electron micromorphology of the epoxy/316L metal lattice composites. (**a**) The SEM of the 316L matrix. (**b**) The SEM of the 316L matrix including some pore defect. (**c**) The SEM of the interface between 316L and epoxy. (**d**) The SEM of the interface between 316L and epoxy including pore defects. (**e**) The SEM of the epoxy with bubble defects. (**f**) The SEM of the epoxy matrix.

Figure 5a,b are the secondary electron micromorphology of the epoxy/316L metal lattice composites. There are no defects such as cracks or incomplete fusion, only small-sized pores. Figure 5c,d show the interface contact between the epoxy and the metal. The interface is generally well bonded, but bubbles are easily generated at the interface, resulting in incomplete bonding of the interface. Figure 5e,f are the molding quality of the epoxy; the overall molding is good, but there are local bubbles that are not exhausted.

Table 1 shows the damping properties of the 316L metallic lattice structure and the epoxy/316L metallic lattice composites. The damping ratio of the 316L metallic lattice structure is 0.99743%, which is much higher than that of the 316L metal matrix [31], which is about 10 times higher than the base, indicating that the structural design can effectively improve damping performance. The porosity of the lattice structure exceeds 50%. Through the filling of epoxy, the pore part is occupied by epoxy, and the damping ratio of the epoxy/316L metallic lattice composites has been greatly improved, reaching 13.31156%.

Epoxy is a viscoelastic material whose dynamic mechanical behavior is different from elastic materials such as steel. The stress and strain of steel are almost simultaneous. However, when the alternating stress is applied, its deformation will obviously lag behind the external force of the materials. When the external force changes, the epoxy will move and convert some mechanical energy into heat energy and dissipate, thereby consuming energy. Meanwhile, the interface between epoxy and metal is a weak bonding interface. The interface is not as tight as metal to metal. When the shear stress of the interface is sufficient to overcome the bonding force of the interface, the interface will effectively consume energy.

It can be concluded that the damping of the composite material includes multiple damping sources: the damping of the metal itself, the damping generated by the lattice structure, the damping of the epoxy itself, and the damping generated by the resin and the metal structure. The final damping performance is the result of the superposition of multiple damping sources.

### 3.2. Mechanical Properties

**Figure 6:**
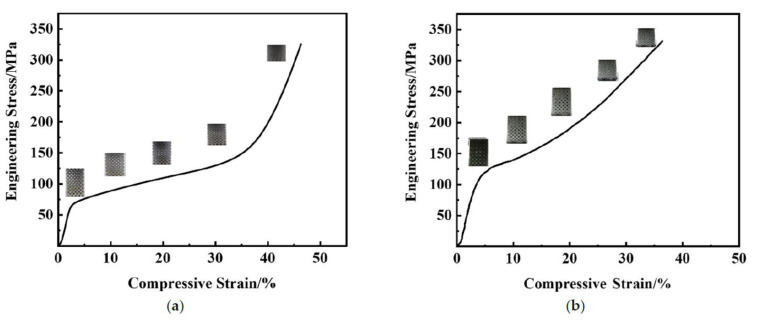
The curves of compressive stress–strain: (**a**) the 316L metallic lattice structure, (**b**) epoxy/316L metallic lattice composites.

Figure 6a is the compressive stress–strain curve of the 316L metallic lattice structure. When an external force is applied to compress, the deformation mode of the 316L metal lattice is uniform deformation, and the height is reduced along the Z axis. At the same time, similar to porous materials, lattice metals also have three distinct characteristic stages. Firstly, in the initial stage of compression, the curve of compressive stress–strain shows obvious linear behavior. Secondly, with the continuous compression, the stress of the material decreases obviously after yielding, and begins to enter the platform area. In this interval, the stress changes little and rises slowly. The pores are finally filled, and the deformation of the material is dominated by the plastic deformation of the metal. Finally, as the strain increases, the pores have been filled and the stress increases significantly, which can be attributed to the large number of dislocations generated during the compression process of the metal material.

**Figure 7:**
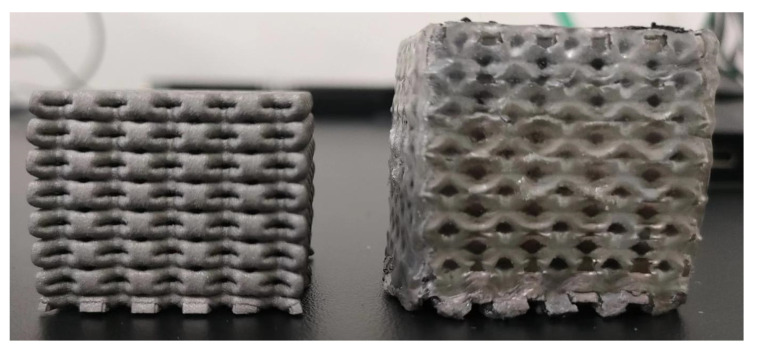
Comparison of 316L metal lattice and epoxy/316L metal lattice composites after compression.

Figure 6b shows the stress–strain curve of the epoxy/316L metallic lattice composite. Unlike the pure lattice structure, the stress required to produce the same strain becomes larger, and the final deformation is about 35%, while the final deformation of the pure lattice structure is about 45%. The final deformation difference is about 10% at the same pressure. As per Figure 7, epoxy/316L lattice composites have better performance under the same pressure. At the same time, it is obvious that epoxy/316L lattice composites have less deformation in the compression direction. Furthermore, the platform area is not obvious. With the increase of strain, the stress increases slowly. The yield strength of the material is improved by filling epoxy into the 316L metallic lattice structure. When the strain continues to increase, the epoxy will begin to be subjected to axial pressure. Epoxy resin has two functions: firstly, it will delay or prevent the deformation and collapse of the hole wall; secondly, it will also lead to lateral deformation of the composite material. The density of epoxy will increase through compression. When the compressive stress is large enough, part of the epoxy will be squeezed out of the 316L metallic lattice structure. Due to the addition of toughening agent in the production of the epoxy/316L metallic lattice structure, the epoxy is not broken due to the increase in stress. Epoxy will be squeezed out in the pores of the lattice metal, but the extrusion process is very slow and only a small part is squeezed out. When the strain increases to a certain extent, the transverse deformation of the filling material in the composite material will further increase. Finally, the metal skeleton of the composite material is squeezed and fractured.

By comparing the stress–strain curves of the compression process, the force required to produce the same deformation increases. For example, when the deformation is 10%, the force required for the 316L metal lattice is 87.56 MPa, while the force required for the epoxy/316L metallic lattice composite as prepared is 140.03 MPa. The prepared composite material greatly enhances the resistance to deformation.

**Figure 8:**
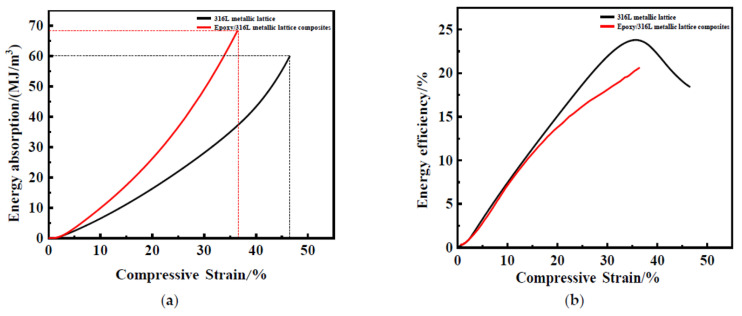
(**a**) Energy absorption curve of the 316L metallic lattice structure and the epoxy/316L metallic lattice composites. (**b**) The curve of energy absorption efficiency.

Figure 8 shows the absorption energy curves of the 316L metallic lattice structure and the epoxy/316L metallic lattice composites. From the beginning of compression to the final densification process, the energy absorbed by the material increases. The energy absorbed by the 316L metallic lattice reaches 59 MJ/m^3^. The energy absorbed by the epoxy/316L metallic lattice structure reaches 68.32 MJ/m^3^, which is significantly higher than that of the pure lattice structure, indicating that the addition of the filler can effectively enhance the energy absorption characteristics of the material.

By comparing the energy absorption efficiency, it can be seen that the energy absorption efficiency of the 316L metallic lattice material increases first and then decreases. The point where the curve derivative is 0 is the end of the platform area, and the lattice structure begins densification at this point. Energy absorption efficiency before this point continues to increase due to the lattice structure. The energy absorption is still increasing after this point due to the 316L metal matrix absorbing energy, but energy absorption efficiency begins to decline. The energy absorption efficiency curve of the epoxy/316L metallic lattice structure is in an increasing state. In the range of 10% strain, the energy absorption efficiency of the two materials is equal, but the energy absorption efficiency is lower at large strains, which may be caused by the epoxy filling.

## 4. Conclusions

316L metallic lattice structure was fabricated by laser selective melting method, and epoxy/316L metallic lattice composite was fabricated by filling epoxy into the pores of the lattice structure. The structural design effectively reduces the weight and has good mechanical properties. The damping performance of the material is improved by the filling of epoxy. At the same time, due to the superposition of multiple damping sources and the filling of pores, the epoxy/316L metallic lattice composites show high damping characteristics, and the damping ratio reaches 13.31156%. At the same time, the designed epoxy resin/316L metallic lattice composite material has good energy absorption characteristics, which can reach 68.32 MJ/m^2^ at the highest.

## Figures and Tables

**Table 1:** Damping properties of fabricated 316L metallic lattice structure and the epoxy/316 metallic lattice composites.

Material	316L	316L Metallic Lattice	Epoxy/316L Metallic Lattice Composites
Damping ratios/%	0.1 [31]	0.99743	13.31156

## Data Availability

Not applicable.

## References

[1] Fu Y., Kabir I.I., Yeoh G.H., Peng Z. (2021). A review on polymer-based materials for underwater sound absorption. Polym. Test..

[2] Katsiropoulos C., Pappas P., Koutroumanis N., Kokkinos A., Galiotis C. (2022). Enhancement of damping response in polymers and composites by the addition of graphene nanoplatelets. Compos. Sci. Technol..

[3] Vervaeke R., Debruyne S., Vandepitte D. (2019). Numerical and experimental analysis of vibration damping performance of polyurethane adhesive in machine operations. Int. J. Adhes. Adhes..

[4] Li M., Liu J., Yan S., Yan W., Shi B. (2020). Effect of aging treatment on damping capacity in Cu-Al-Mn shape memory alloy. J. Alloys Compd..

[5] Sun M., Jiang Y., Wang X., Zhang L., Jiang W., Liu R., Wang H., Kong M., Gao Y., Hao T. (2019). Synchronously enhanced mechanical and damping properties of Fe-18Ga alloy by mechanical treatment. Mater. Sci. Eng. A.

[6] Song M., Yue X., Wang X., Huang M., Ma M., Pan W., Qin Q. (2020). Improved high-temperature damping performance of nitrile-butadiene rubber/phenolic resin composites by introducing different hindered amine molecules. e-Polymers.

[7] Zhao M., Shao Y., Zheng W., Luo Y., Qiao J., Wu S., Yan Y., Guo W. (2021). Tailoring the damping and mechanical properties of porous NiTi by a phase leaching process. J. Alloys Compd..

[8] Zheng N. (2020). Fabrication and damping behaviors of novel polyurethane/TiNiCu composites. Phys. B.

[9] Ji X., Wang Q., Yin F., Cui C., Ji P., Hao G. (2018). Fabrication and properties of novel porous CuAlMn shape memory alloys and polymer/CuAlMn composites. Compos. Part A.

[10] Wang Q., Wang L., Kang J., Wang Q., Cui C., Su R., Narayanaswamy B. (2020). Effects of aging and thermal cycling on the microstructure and damping behaviors of a porous CuAlMn shape memory alloy. J. Mater. Res. Technol..

[11] Wang Q., Liu X., Li B., Yin F., Cui C., Ding Y., Jiao Z. (2018). Fabrication and damping behavior of a novel Mg/TiNiCu composite. Mater. Lett..

[12] Yang K., Yang X., He C., Liu E., Shi C., Ma L., Li Q., Li J., Zhao N. (2017). Damping characteristics of Al matrix composite foams reinforced by in-situ grown carbon nanotubes. Mater. Lett..

[13] Dahil L., Karabulut A., Baspinar S. (2013). Damping Properties of open pore aluminum foams produced by vacuum casting and NaCI dissolution process. Metalurgija.

[14] Chen M., Liu B., Ji Z., Jia C., Wu Q., Liu Z. (2020). Mechanical properties and damping properties of carbon nanotube-reinforced foam aluminum with small aperture. J. Mater. Res..

[15] Liao X., Wang Y., Fan G., Liu E., Shang J., Yang S., Luo H., Song X., Ren X., Otsuka K. (2017). High damping capacity of a Ni-Cu-Mn-Ga alloy in wide ambienttemperature range. J. Alloys Compos..

[16] Matli P.R., Manakari V., Parande G., Mattli M.R., Shakoor R.A., Gupta M. (2020). Improving Mechanical, Thermal and Damping Properties of NiTi(Nitinol) Reinforced Aluminum Nanocomposites. J. Compos. Sci..

[17] Xie B., Yan S., Zou Q. (2016). Facile synthesis of carbon nanotubes on nickel foam by decomposition of acetylene in chemical vapor deposition. J. Nanosci. Nanotechnol..

[18] Wang Y., Yao D., Zheng Y. (2022). Effect of graphene size on the mechanical and damping properties of polyether amine modified r-GO and ZnO multi-nanoparticles filled epoxy. Mater. Lett..

[19] Mallipudi P.K., Jyothi P., Ramanaiah N., Bhaskara Raju V.V.S. (2021). Damping Performance of Polychloroprene Rubber for Unconstrained Damping Applications. Adv. Sci. Technol..

[20] Zhang W., Ma F., Meng Z., Kong L., Dai Z., Zhao G., Zhu A., Liu X.Y., Lin N. (2021). Green Synthesis of Waterborne Polyurethane for High Damping Capacity. Macromol. Chem. Phys..

[21] Song M., Yue X.L., Wang X.J., Cao F.Y., Li Y.N., Su C.H., Qin Q. (2020). Effect of Hindered Phenol AO-80 on the Damping Properties for Nitrile-Butadiene Rubber/Phenolic Resin: Molecular Simulation and Experimental Study. Macromol. Mater. Eng..

[22] Azammi A.N., Sapuan S., Ishak M.R., Sultan M.T. (2020). Physical and damping properties of kenaf fibre filled natural rubber/thermoplastic polyurethane composites. Def. Technol..

[23] Shi X., Liu C., Li K., Shi Z., Cui Z. (2019). Effect of microcapsules partially filled with viscoelastic acrylate polymer on damping behaviours of epoxy resin. New J. Chem..

[24] Zhou R., Gao W., Xia L., Wu H., Guo S. (2018). The study of damping property and mechanism of thermoplastic polyurethane/phenolic resin through a combined experiment and molecular dynamics simulation. J. Mater. Sci..

[25] Wang Y., Zhan M., Li Y., Shi M., Huang Z. (2012). Mechanical and Damping Properties of Glass Fiber and Mica-Reinforced Epoxy Composites. Polym. Plast. Technol. Eng..

[26] Yu Y.H., Wu X.N., Xu P. (2010). Research on Damping of Foamed Al Composite Filled Epoxy Resin in the Holes. Adv. Mater. Res..

[27] Zhan C., Li M., McCoy R., Zhao L., Lu W. (2022). 3D printed hierarchical re-entrant honeycombs: Enhanced mechanical properties and the underlying deformation mechanisms. Compos. Struct..

[28] Zhang M., Yu Q., Liu Z., Zhang J., Tan G., Jiao D., Zhu W., Li S., Zhang Z., Yang R. (2020). 3D printed Mg-NiTi interpenetrating-phase composites with high strength, damping capacity, and energy absorption efficiency. Sci. Adv..

[29] Ouyang D., Xing W., Li N., Li Y., Liu L. (2018). Structural evolutions in 3D-printed Fe-based metallic glass fabricated by selective laser melting. Addit. Manuf..

[30] Lu H.Z., Ma H.W., Cai W.S., Luo X., Qu S.G., Wang J., Lupoi R., Yin S., Yang C. (2022). Altered phase transformation behaviors and enhanced bending shape memory property of NiTi shape memory alloy via selective laser melting. J. Mater. Process. Tech.

[31] Wei Y., Yu B., Yang Q., Gao P., Miao Z., Cheng J., Sun X. (2020). Damping behaviors of steel-based Kelvin lattice structures fabricated by indirect additive manufacture combining investment casting. Smart Mater. Struct..

[32] Uhríčik M., Oravcová M., Palček P., Oršulová T., Hanusová P. (2019). Analysis of dependence of internal damping on temperature of austenitic stells AISI 304 and 316L. Res. Procedia.

